# Decline in in-patient treatments of genital warts among young Australians following the national HPV vaccination program

**DOI:** 10.1186/1471-2334-13-140

**Published:** 2013-03-18

**Authors:** Hammad Ali, Rebecca J Guy, Handan Wand, Tim RH Read, David G Regan, Andrew E Grulich, Christopher K Fairley, Basil Donovan

**Affiliations:** 1 The Kirby Institute, The University of New South Wales, Sydney, NSW, Australia; 2 Melbourne Sexual Health Centre, Melbourne, VIC, Australia; 3 School of Population Health, University of Melbourne, Melbourne, VIC, Australia; 4 Sydney Sexual Health Centre, Sydney Hospital, Sydney, NSW, Australia

## Abstract

**Background:**

There has been a rapid decline in the number of young heterosexuals diagnosed with genital warts at outpatient sexual health services since the national human papillomavirus (HPV) vaccination program started in Australia in 2007. We assessed the impact of the vaccination program on the number of in-patient treatments for genital warts.

**Methods:**

Data on in-patient treatments of genital warts in all private hospitals were extracted from the Medicare website. Medicare is the universal health insurance scheme of Australia. In the vaccine period (2007–2011) and pre-vaccine period (2000–2007) we calculated the percentage change in treatment numbers and trends in annual treatment rates in private hospitals. Australian population data were used to calculate rates. Summary rate ratios of average annual trends were determined.

**Results:**

Between 2000 and 2011, 6,014 women and 936 men aged 15–44 years underwent in-patient treatment for genital warts in private hospitals. In 15–24 year old women, there was a significant decreasing trend in annual treatment rates of vulval/vaginal warts in the vaccine period (overall decrease of 85.3% in treatment numbers from 2007 to 2011) compared to no significant trend in the pre-vaccine period (summary rate ratio (SRR) = 0.33, p < 0.001). In 25–34 year old women, declining trends were seen in both vaccine and pre-vaccine periods (overall decrease of 33% vs. 24.3%), but the rate of change was greater in the vaccine period (SRR = 0.60, p < 0.001). In 35–44 year old women, there was no significant change in both periods (SRR = 0.91, p = 0.14). In 15–24 year old men, there was a significant decreasing trend in annual treatment rates of penile warts in the vaccine period (decrease of 70.6%) compared to an increasing trend in the pre-vaccine period (SRR = 0.76, p = 0.02). In 25–34 year old men there was a significant decreasing trend in the vaccine period compared to no change in the pre-vaccine period (SRR = 0.81, p = 0.04) and in 35–44 year old men there was no significant change in rates of penile warts both periods, but the rate of change was greater in the vaccine period (SRR = 0.70, p = 0.02).

**Conclusions:**

The marked decline in in-patient treatment of vulval/vaginal warts in the youngest women is probably attributable to the HPV vaccine program. The moderate decline in in-patient treatments for penile warts in men probably reflects herd immunity.

## Background

Australia became the first country to introduce a national quadrivalent human papillomavirus (HPV) vaccine (Gardasil, CSL Biotherapies, Melbourne, VIC, Australia) program for young women in mid 2007 [1]. The ongoing voluntary program provides free vaccine to 12–13 year old girls and there was a catch-up program for women up to 26 years from 2007 to 2009. In addition to providing protection against HPV 16 and 18, the quadrivalent vaccine provides protection against HPV 6 and 11 which causes more than 90% of genital warts [2]. Vaccine coverage rates have been reported to be very high in Australia with >80% coverage rates for the first dose and ~70% for the three doses in 12–17 year old girls [1]. Lower coverage rates have been reported for women aged 18–19 years (64%) and 20–26 years (52%) for the first dose [1].

Before the vaccination program started, genital warts were the most common condition diagnosed at sexual health services in Australia [3]. Since then, sentinel surveillance data has shown a large decrease in the proportion of vaccine-eligible women diagnosed with genital warts at out-patient sexual health services nationwide [4,5]. In addition, data from the Victorian Cervical Cytology Registry has shown a significant decrease in the incidence of high-grade cervical abnormalities in girls younger than 18 years since the vaccine program started [6].

Approximately 7% of genital warts cases end in hospitalisations [7] and these are an important outcome to monitor when assessing the impact of a public health program due to the severity and associated cost. In-patient treatment under anaesthesia is usually reserved for the most severe or intractable cases of genital warts. The procedure is unpleasant for patients and expensive for the health care system [7]. Genital warts can be prolonged or recurrent and have a significant impact on the quality of life of the patient [8]. In addition, psychosexual vulnerability (including depression, anxiety and anger) has been shown to increase with the number of recurrences [9].

In this study, we assess for the first time the impact of the national HPV vaccination program on private in-patient treatments for genital warts.

## Methods

### Study design

We conducted a time-series analysis using data from a national registry reporting numbers of patients treated under anaesthesia for genital and anal warts in all private hospitals in Australia.

### Data source

Medicare is the universal health insurance scheme of Australia and rebates services provided by private doctors and laboratories. Services provided in the public sector are funded by the state and territory governments and are not rebated by Medicare. Each service has a unique item number [10] and aggregated data are publicly available from the Medicare registry (the Medicare Benefit Schedule website [11]).

We extracted data on in-patient treatments under general anaesthesia or regional or field nerve block (excluding pudendal block) requiring admission to a hospital, among 15–44 year olds from 2000 to 2011. The data were aggregated by 6-month time periods, sex, age-group (15–24 years, 25–34 years and 35–44 years), and anatomical site (vulval/vaginal warts - Medicare item numbers 35507, 35508; penile warts - Medicare item number 36815; anal warts - Medicare item numbers 32177, 32180).

### Statistical analyses

In the vaccine period (2007–2011) and pre-vaccine period (2000–2007) we conducted a descriptive analysis of the number of in-patient treatments per year stratified by sex, age-group and anatomical site and calculated percentage change in treatment numbers. We also calculated annual treatment rates per 100,000 populations. Australian population data (derived from the Australian Bureau of Statistics [12]) were used to calculate rates.

We used Box-Jenkins [13] time-series methodology to determine average annual trends in rates of in-patient treatments. Residuals from the time-series models were examined by autocorrelation and partial autocorrelation methods to detect potential serial dependence in the data. When significant serial dependence was detected, Newey-West [14] autocorrelation and heteroscedasticity-corrected standard errors were used in the relevant regression model assuming that the count data (i.e. number of diagnoses per year) followed a Poisson distribution. Estimated models were considered most appropriate if they typically simulated historical behaviour.

Finally, we compared the average annual treatment rates in the pre-vaccine and vaccine periods and describe summary rate ratios along with 95% confidence intervals.

Analyses were conducted and all models were fitted using STATA v12.1 (StataCorp, Texas, US).

## Results

### Vulval/vaginal warts in women

**Figure 1:**
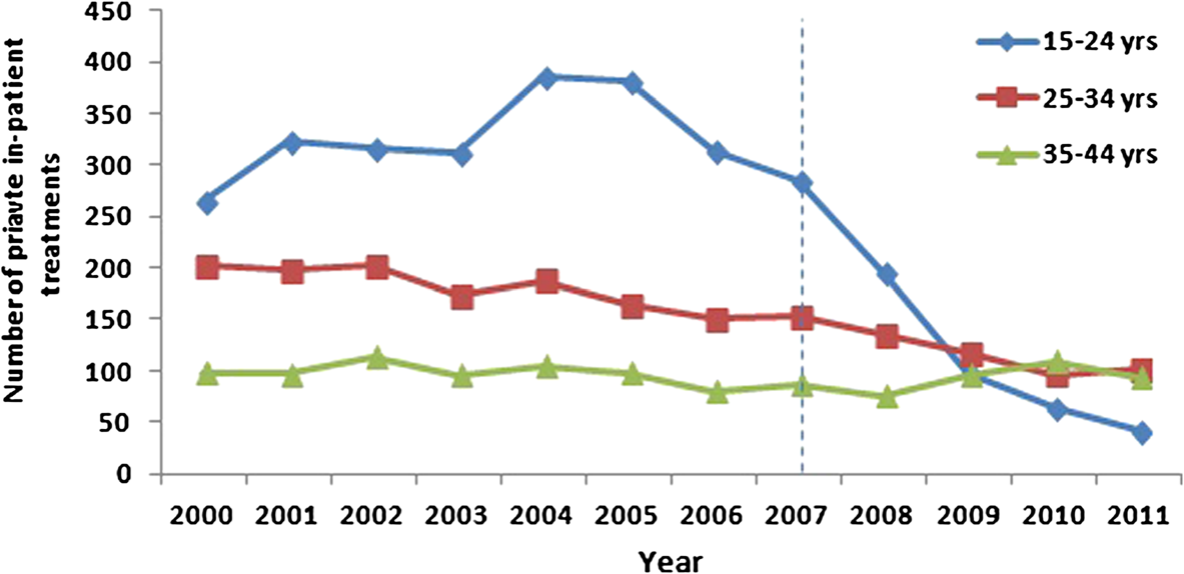
Numbers of in-patient treatments for vulval/vaginal warts in women by age-group, 2000–2011.

Between 2000 and 2011, a total of 6,014 women aged 15–44 years underwent in-patient treatment for vulval/vaginal warts. In 15–24 year old women in the pre-vaccine period the number of treatments did not change (266 treatments in 2000 and 285 in 2007) and there was no significant trend in treatment rates (p = 0.56). In the vaccine period the number of treatments declined by 85.3% to 42 treatments in 2011 (p < 0.001) (Figure 1). The summary rate ratio in the vaccine versus pre-vaccine period in 15–24 year olds was 0.33 (p < 0.001) (Table 1).

**Table 1:** Average annual trends in the numbers of in-patient treatment of warts with summary rate ratios, 2000–2011

	**Pre-vaccine period**			**Vaccine period**			**Pre-vaccine vs vaccine period**		
	**Average annual trend**	**95% CI**	**p-value**	**Average annual trend**	**95% CI**	**p-value**	**Summary rate ratio**	**95% CI**	**p-value**
**Female – vulval/vaginal**									
**15-24 years**	1.00	0.99 - 1.01	0.556	0.76	0.73 - 0.79	<0.001	0.33	0.30 - 0.37	<0.001
**25-34 years**	0.98	0.97 - 0.99	0.001	0.93	0.90 - 0.97	<0.001	0.60	0.54 - 0.66	<0.001
**35-44 years**	0.99	0.97 - 1.00	0.116	1.03	0.99 - 1.07	0.115	0.91	0.81 - 1.03	0.145
**Male – penile**									
**15-24 years**	1.1	1.04 - 1.1	<0.001	0.86	0.80 - 0.93	<0.001	0.76	0.62 - 0.96	0.022
**25-34 years**	0.98	0.95 - 1.01	0.144	0.90	0.84 - 0.96	0.001	0.81	0.66 - 0.99	0.040
**35-44 years**	0.97	0.93 - 1.01	0.125	0.93	0.84 - 1.03	0.161	0.70	0.51 - 0.95	0.024
**Male – anal**									
**15-24 years**	1.08	1.03 - 1.14	0.003	0.90	0.81 - 1.00	0.055	0.92	0.77 - 1.10	0.370
**25-34 years**	0.97	0.94 - 1.01	0.143	0.95	0.87 - 1.04	0.241	0.69	0.59 - 0.79	<0.001
**35-44 years**	0.99	0.95 - 1.03	0.559	0.91	0.83 - 0.99	0.027	0.86	0.74 - 0.99	0.036

CI = confidence interval.

Among women aged 25–34 years, the number of treatments decreased by 24.3% in the pre-vaccine period from 202 in 2000 to 153 in 2007 (p = 0.001) and decreased by 33% to 102 in-patient treatments in 2011 in the vaccine period (p < 0.001). The summary rate ratio in vaccine vs pre-vaccine period in 25–34 year olds was 0.60 (p < 0.001).

Among women aged 35–44 years, there was no significant trend either in the pre-vaccine (98 treatments in 2000 and 87 in 2007; p = 0.12) or vaccine period (94 treatments in 2011; p = 0.11). The summary rate ratio in vaccine vs pre-vaccine period in this age group was 0.91 (p = 0.14).

### Penile warts in men

**Figure 2:**
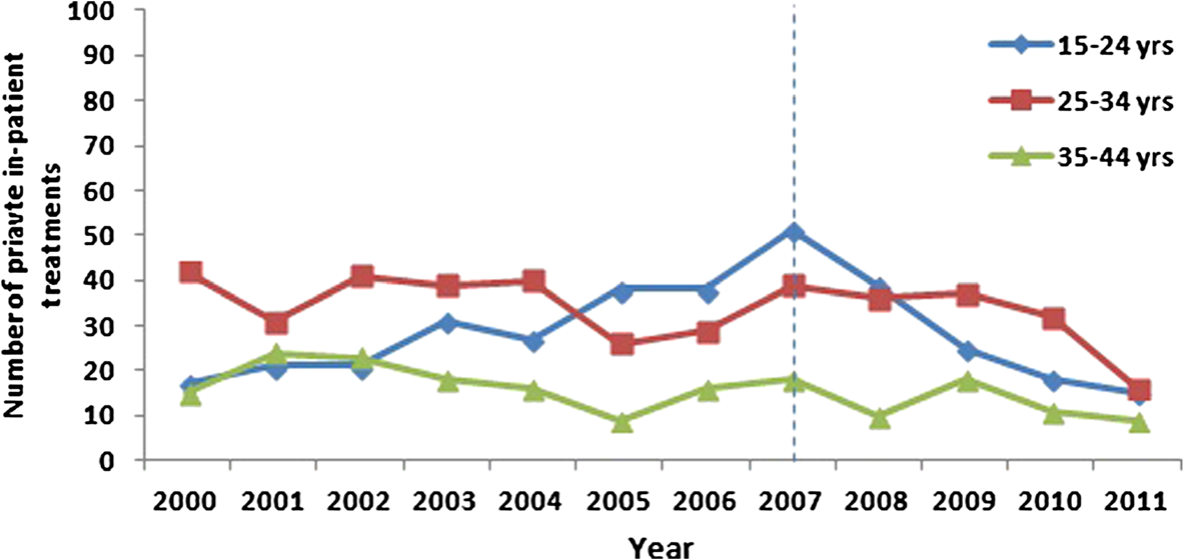
Numbers of in-patient treatments for penile warts in men by age-group, 2000–2011.

Between 2000 and 2011, a total of 936 men aged 15–44 years underwent in-patient treatment for penile warts. The number of 15–24 year old men undergoing in-patient treatment for penile warts in the pre-vaccine period increased by 200% from 17 in 2000 to 51 in 2007 (p < 0.001) and in the vaccine period decreased by 70.6% to 15 treatments in 2011 (p < 0.001) (Figure 2). In this youngest age-group, the summary rate ratio in the vaccine vs pre-vaccine period was 0.76 (p = 0.02) (Table 1).

In the pre-vaccine period, there was no significant trend in in-patient treatments in 25–34 year old (42 in treatments 2000 and 39 in 2007; p = 0.14) or 35–44 year old men (15 in 2000 to 18 in 2007; p = 0.12). In the vaccine period the number of treatments declined by 59.0% to 16 in 2011 in 25–34 year olds (p = 0.001) and there was no significant trend in treatments in 35–44 year old men (p = 0.16). The summary rate ratio in vaccine vs pre-vaccine period was 0.81 (p = 0.04) for the 25–34 year olds and 0.70 (p = 0.02) for 35–44 year olds.

### Anal warts in men

**Figure 3:**
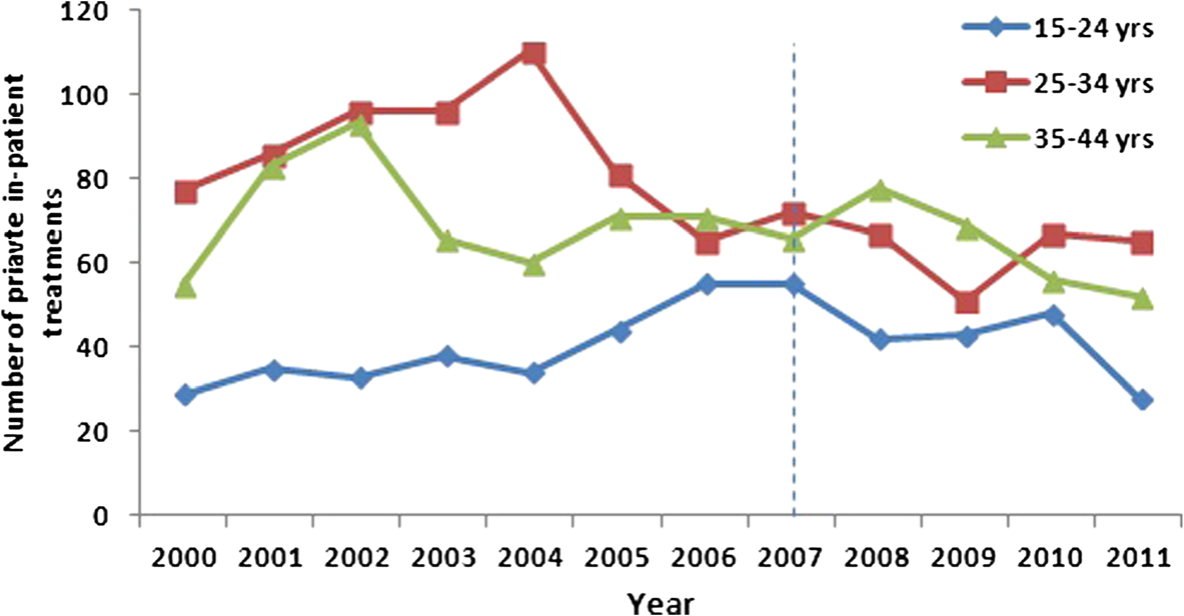
Numbers of in-patient treatments for anal warts in men by age-group, 2000–2011.

Between 2000 and 2011, a total of 2,237 men aged 15–44 years underwent in-patient treatment for anal warts. The number of 15–24 year old men undergoing in-patient treatment for anal warts in the pre-vaccine period increased by 89.6% from 29 in 2000 to 55 in 2007 (p = 0.003) and in the vaccine period the numbers decreased by 49.1% to 28 treatments in 2011 (p = 0.05) (Figure 3). In this youngest age-group, the summary rate ratio for in-patient treatments of anal warts in vaccine vs pre-vaccine period was 0.92 (p = 0.37) (Table 1).

Among men aged 25–34 years, there was no significant trend either in the pre-vaccine (77 treatments in 2000 and 71 in 2007; p = 0.14) or vaccine period (65 treatments in 2011; p = 0.24). The summary rate ratio in vaccine vs pre-vaccine period in this age group was 0.69 (p < 0.001). There was no significant trend in the number of in-patient treatments among 35–44 year old men in the pre-vaccine period (55 treatments in 2000 to 66 in 2007; p = 0.56). In the vaccine period there was a 21.2% decline in number of treatments to 52 in 2011 (p = 0.03). The summary rate ratio in vaccine vs pre-vaccine period was 0.86 (p = 0.04).

## Discussion

This is the first study to look at the impact of the HPV vaccine program on in-patient treatment of genital warts in Australia. We found that there was a large (85.3%) decline in the number of in-patient treatments for genital warts in the youngest (15–24 year old) women after the HPV vaccination program began in 2007; the decline in the 25–34 year old age-group was more modest and there was no significant decline in the numbers of in-patient treatments in older (35–44 year old) women. There were also moderate declines in the numbers of treatments for penile warts in 15–24 and 25–34 year old men and for anal warts in 35–44 year old men.

The major strength of this analysis is the use of nation-wide population-based data and a long follow-up time, providing a complete picture of the private in-patient treatment of genital and anal warts in Australia. Use of a national register eliminates the problem of recall bias from self-reported information. The major limitation of this study is that Medicare data only represents treatments in private hospitals and does not include treatments in publicly funded hospitals; thus the data are not representative of all in-patient treatments. Another limitation of the study is that the hospital data extracted may contain repeat in-patient treatments for the same individual but numbers would be limited and we do not anticipate this pattern to have changed during the study period. Lastly, as sexual behaviour data were not available we were unable to adjust for any change in sexual risk behaviour – a decline for example could have contributed to decreasing trends observed. However, national surveys show that sexual risk taking behaviour has increased in adolescents [15] and gay men [16] in the vaccine period compared to pre-vaccine period and these changes have been associated with an increase in the prevalence of chlamydia in both populations [17,18].

A number of factors could potentially influence the trends observed. Firstly, socio-economic status of a person or a family could theoretically affect both going to private hospitals and the uptake of vaccine. However, we don’t believe the vaccine uptake would vary considerably by socio-economic status as the HPV vaccination program in Australia provides vaccines free of charge to all young women, and boasts high coverage rates (>80% in school-girls [1]). Second, a decline in the proportion of the population with insurance coverage over time could explain the trends observed. However, data from the Australian Private Health Insurance Administration Council shows that there was a significant increase in the proportion of population which have hospital treatment insurance coverage; from 45.4% in Dec 2000 to 46.3% in Dec 2011 (p < 0.001) [19]. Third, any changes in healthcare-seeking behaviour or clinical practices could also influence the declining trends observed. It is possible that an increase in the use of self-applied topical treatment of genital warts over time may have contributed to the decline in the surgical treatments seen in our analysis. However, topical treatments were available throughout the study period and the price did not change substantially over time. Furthermore, if there was a decline due to increase in the use of topical treatments we would expect it to have been seen in all age-groups, thus the decline observed only in young people cannot be explained by changes in the topical treatment patterns. Lastly, the national incidence of genital warts has been reported to be 2.2 per 1,000 persons (2.1 in males and 2.3 in females) in the pre-vaccine period [7], thus the few hundred cases of genital warts treated in the private hospitals is a relatively small proportion compared to ~45,000 cases of genital warts in 2006 nationally. However, this is an important proportion of clients as mainly chronic and/or severe cases are likely to be referred to a hospital for in-patient treatment (surgery under anaesthesia is not recommended as a first-line therapy for the treatment of genital warts [20] and cannot be performed in a general practice or sexual health clinic setting).

The results from our study validate findings from a national sentinel surveillance system at sexual health services [4,5] and confirm that the numbers of cases of genital warts are declining in young women since the vaccine roll out (the 15–24 year old women in our study were all eligible for free HPV vaccination between 2007 and 2009). We believe that the smaller decline in 25–34 year old women is because only a proportion of these women were vaccinated as part of the initial vaccine catch-up program [1]. Men were not eligible for the free HPV vaccination and thus the decline in penile warts in men after 2007 is likely to reflect herd immunity, as observed in the sentinel surveillance system [4]. Our study also showed that a higher number of women underwent inpatient treatment compared to men. That could be because penile warts are easier to treat in the out-patient setting (and to self-treat), compared to vulval/vaginal warts.

We also reported on the numbers of treatment of anal warts in men as a comparison. There was no decline in the number of treatments for anal warts in the younger men after 2007. The moderate decline in older men in the vaccine period could also reflect herd immunity as some men undergoing treatment for anal warts were likely be heterosexual or bisexual. In one study 18.2% of men with anal warts were heterosexuals [21]. A recent review [22] of anal sexual practices in heterosexuals highlighted the paucity of data in this area including sexual practices such as anal digital stimulation of men by their female partner. Ongoing monitoring of the impact of the vaccine in men is warranted as the Australian Government has extended the national HPV vaccination program to include vaccination for boys in 2013 [23].

## Conclusion

In conclusion, this study has confirmed the utility of using readily available in-patient treatment data for monitoring the impact of the HPV vaccine program. Further efforts should be made to establish systems to collate and report public hospital separations data on genital warts treatment. Collation of a range of data sources is warranted for countries about to roll out the quadrivalent HPV vaccine including: vaccine coverage, vaccine safety, surveillance, cervical cytology coverage, incidence of cervical cancer, genital warts and recurrent respiratory papillomatosis, treatment data on cervical cancer and genital warts and knowledge, attitudes and beliefs about HPV and HPV vaccination [24].

### Key messages

• Between 2007 and 2011, the number of in-patient treatments for vulval/vaginal warts declined by 85.3% in 15–24 year old women, confirming the population effect of the HPV vaccine program.

• In the same time period, there was a more modest decline in the number of in-patient treatments for penile warts in men, suggesting herd-immunity.

## Competing interests

CKF owns shares in CSL Biotherapies. CKF, AEG, DGR, RJG, and BD have received honoraria from CSL Biotherapies. BD and RJG have received honoraria from Sanofi Pasteur MSD. CFK, DGR, AEG, RJG and BD receive research funding from CSL Biotherapies. BD, CFK and AEG have received honoraria from Merck. AEG sits on the Australian advisory board for the Gardasil vaccine. TR is a site investigator in a Merck vaccine study.

## Authors’ contributions

BD, RJG and HA conceptualized the study. HA extracted the data. HA and HW conducted the analysis. HA wrote the first draft. BD, RJG, TRHR, DJR, AEG and CKF advised on analysis and interpretation. All authors read and approved the final manuscript.

## Pre-publication history

The pre-publication history for this paper can be accessed here:

http://www.biomedcentral.com/1471-2334/13/140/prepub
